# Protective Effect of Resveratrol against Hexavalent Chromium-Induced Genotoxic Damage in Hsd:ICR Male Mice

**DOI:** 10.3390/molecules27134028

**Published:** 2022-06-23

**Authors:** Tonancy Nicolás-Méndez, Sam Kacew, Alda Rocío Ortiz-Muñiz, Víctor Manuel Mendoza-Núñez, María del Carmen García-Rodríguez

**Affiliations:** 1 Laboratorio de Antimutagénesis, Anticarcinogénesis y Antiteratogénesis Ambiental, Facultad de Estudios Superiores—Zaragoza, Universidad Nacional Autónoma de México (UNAM), Mexico City 09230, Mexico; tonic_1986@comunidad.unam.mx; 2 Posgrado en Ciencias Biológicas, Universidad Nacional Autónoma de México (UNAM), Mexico City 04510, Mexico; 3 McLaughlin Centre for Population Health Risk Assessment, University of Ottawa, Ottawa, ON K2G 3G8, Canada; skacew@uottawa.ca; 4 Departamento de Ciencias de la Salud, Universidad Autónoma Metropolitana (UAM), Mexico City 09310, Mexico; arom@xanum.uam.mx; 5 Unidad de Investigación en Gerontología, Facultad de Estudios Superiores—Zaragoza, Universidad Nacional Autónoma de México (UNAM), Mexico City 09230, Mexico; mendovic@unam.mx

**Keywords:** resveratrol, hexavalent chromium, 8-hydroxydeoxyguanosine adduct repair, apoptosis, endogenous antioxidant system, antigenotoxic

## Abstract

The aim of this study is to examine the ability of resveratrol to counteract hexavalent chromium [Cr(VI)]-induced genetic damage, as well as the possible pathways associated with this protection. Hsd:ICR male mice are divided into groups of the following five individuals each: (a) control 1, distilled water; (b) control 2, ethanol 30%; (c) resveratrol, 50 mg/kg by gavage; (d) CrO_3_, 20 mg/kg intraperitoneally; (e) resveratrol + CrO_3_, resveratrol administered 4 h prior to CrO_3_. The assessment is performed on peripheral blood. Micronuclei (MN) kinetics are measured from 0 to 72 h, while 8-hydroxydeoxyguanosine (8-OHdG) adduct repair levels, endogenous antioxidant system biomarkers, and apoptosis frequency were quantified after 48 h. Resveratrol reduces the frequency of Cr(VI)-induced MN and shows significant effects on the 8-OHdG adduct levels, suggesting that cell repair could be enhanced by this polyphenol. Concomitant administration of resveratrol and Cr(VI) results in a return of the activities of glutathione peroxidase and catalase to control levels, accompanied by modifications of superoxide dismutase activity and glutathione levels. Thus, antioxidant properties might play an important role in resveratrol-mediated inhibition of Cr(VI)-induced oxidant genotoxicity. The increase in apoptotic cells and the decrease in necrosis further confirmed that resveratrol effectively blocks the actions of Cr(VI).

## 1. Introduction

Resveratrol (3,4′,5-trihydroxy-trans-stilbene) is a polyphenol found in a group of stilbenes. It has high antioxidant potential associated with beneficial health effects in the context of neurodegenerative and cardiovascular diseases, as well as some types of cancer, diabetes, and obesity-related disorders [1,2]. The antioxidant effects of resveratrol have been attributed to its ability to scavenge reactive oxygen species (ROS), activate repair mechanisms, and induce apoptosis [3,4]. Notably, resveratrol has been found to prevent DNA damage [5,6]. Although the effects of resveratrol on toxicity induced by metals (e.g., arsenic trioxide, sodium arsenite, copper oxide, chromic chloride, and potassium dichromate) have been examined in rodent hearts, livers, kidneys, thymus, and ovaries [7,8,9,10,11], there are no studies evaluating the effects of this polyphenol on hexavalent chromium [Cr(VI)] compound-induced genotoxicity.

Cr(VI) is largely released into the environment due to industrial activities, mainly including electroplating, welding, leather tanning, and pigment manufacturing, or found in automobile exhaust and tobacco products [12,13]. Both acute and chronic exposure to Cr(VI) compounds have been associated with cancer induction in different organs and tissues [14,15]. The genotoxic damage produced during its intracellular reduction may initiate and promote Cr(VI)-induced carcinogenesis by the formation of DNA adducts, cross-linking (DNA-protein and DNA-DNA), abasic sites, and oxidized DNA bases [16]. It is important to highlight that the induction of apoptosis, the inhibition of repair mechanisms, and gene expression play a crucial role in the genotoxic damage generated by exposure to Cr(VI) compounds [15,17]. Several studies have shown that antioxidants can counteract the effects of ROS and free radicals [18], such that antioxidant-rich substances have emerged as potential agents for preventing and adjuvating oxidative stress and DNA damage [19,20,21]. More precisely, compounds such as polyphenols have been shown to play a direct role as radical scavengers and metal chelators and to exert indirect effects by modulating levels of transcription factors and enzymes [22,23,24]. Therefore, the aim of the present study was to examine the protective effects of resveratrol against Cr(VI)-induced genotoxicity in vivo and the underlying metabolic processes including 8-hydroxydeoxyguanosine (8-OHdG, 7,8-dihydro-8-oxodeoxyguanosine) adduct repair, the endogenous antioxidant component system, and apoptosis, which may be involved in preventing Cr(VI)-induced DNA damage.

## 2. Results

### 2.1. Effect of Resveratrol on MN Induced by CrO_3_

**Figure 1:**
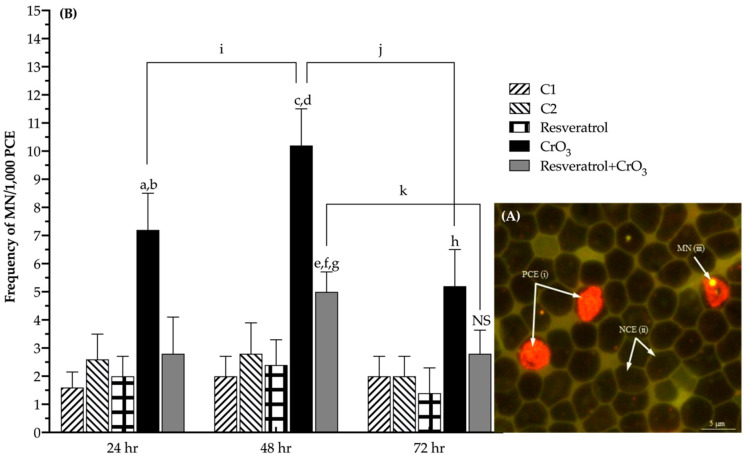
Effect of resveratrol and CrO_3_ on the frequency of micronuclei (MN) were evaluated in the peripheral blood of mice. (**A**) Fluorescent microphotograph (1000×) of peripheral blood cells using the AO coating method. Polychromatic erythrocytes (PCE) stain fluorescent orange (**i**), normochromatic erythrocytes (NCE) do not stain at all (shadow) (**ii**), and MN fluoresces yellow (**iii**). (**B**) Data show the MN frequency at 24, 48, and 72 h minus the MN frequency at 0 h (net induction frequency: NIF, see results text). The frequencies of MN in the resveratrol + CrO_3_ group decreased by 63, 50, and 47% at 24, 48, and 72 h, respectively, compared with those in the CrO_3_ group. A total of 4000 PCE were evaluated in each mouse (*n* = 5 mice/group). Statistical significance was determined using two-way repeated measures-ANOVA followed by Tukey’s post-hoc test. Analysis by treatments: ^a^ *p* < 0.001 vs. C1, 24 h; ^b^ *p* < 0.004 vs. resveratrol + CrO_3_, 24 h; ^c^ *p* < 0.0001 vs. C1, 48 h; ^d^ *p* < 0.001 vs. resveratrol + CrO_3_, 48 h; ^e^ *p* < 0.001 vs. C1, 48 h; ^f^ *p* < 0.040 vs. C2, 48 h; ^g^ *p* < 0.006 vs. resveratrol, 48 h; ^h^ *p* < 0.015 vs. C1, 72 h; ^NS^ *p* > 0.05 vs. CrO_3,_ 72 h. Analysis by time of evaluations: ^i^ *p* < 0.038, CrO_3_; ^j^ *p* < 0.004, CrO_3_; ^k^ *p* < 0.009, resveratrol + CrO_3_. C1, Control 1, vehicle only (distilled water). C2, Control 2, vehicle only (ethanol 30%). CrO_3_, chromium trioxide. AO, acridine orange.

The genotoxic damage caused by Cr(VI) was evaluated using the micronuclei (MN) assay in erythrocytes of peripheral blood using acridine orange (AO)-coated slides. Differential AO staining distinguished polychromatic erythrocytes (PCE) from normochromatic erythrocytes (NCE) because PCE were stained, showing orange fluorescence due to the presence of ribosomal RNA (Figure 1A(i)), while NCE did not stain at all (shadow) (Figure 1A(ii)). The AO also enabled the identification of MN, which exhibited yellow fluorescence due to their DNA content (Figure 1A(iii)). To compare the kinetics of MN induction in treatment groups, data were analyzed by calculating the net induction frequency (NIF) using Equation (1) as follows:

NIF = |MN frequencies measured at *time*
*x_i_* − MN frequencies measured at *time 0*|/*n* (1)

where *x_i_* is the evaluation at 24, 48, or 72 h per group, *time 0* is the evaluation at 0 h (before treatment) per group, and *n* is the number of mice per group.

Calculating the NIF enhanced the ability to determine net MN induction by eliminating baseline MN variability among treated groups at *time 0*. Figure 1B illustrates the NIF of MN values for all treatments at 24, 48, and 72 h after administration. Treatments had a significant effect (*p* < 0.0001) on the MN frequencies according to the two-way repeated-measures analysis of variance (RM-ANOVA). In the chromium trioxide (CrO_3_) group, an increase of about 7, 10, and 5 MN was observed at 24, 48, and 72 h, respectively, which was significantly higher than the control C1 (*p* < 0.001, *p* < 0.0001, and *p* < 0.015, respectively). The group treated with resveratrol and CrO_3_ (resveratrol + CrO_3_) had lower MN frequency than the CrO_3_ only treatment at all times examined and was highest at 48 h (*p* < 0.001), though this reduction was no longer significant at 72 h (*p* > 0.05). However, in this group, the MN frequencies observed at 48 h were significantly different from the control groups (C1, *p* < 0.001; C2, *p* < 0.040) and the group treated with resveratrol alone (*p* < 0.006). There was a significant effect of time on the frequency of MN in the two-way RM-ANOVA (*p* < 0.0001). In the CrO_3_ group, the frequency of MN increased at 48 h with respect to the initial time (*p* < 0.038) and decreased at 72 h (*p* < 0.004). In the resveratrol + CrO_3_ group, the MN frequency decreased at 72 h (*p* < 0.009), and values at the initial and final times were similar. Treatment with resveratrol alone did not significantly affect the frequency of MN compared to control group C2 (*p* > 0.05).

### 2.2. Effect of Resveratrol and CrO_3_ on 8-OHdG Adduct Levels

**Figure 2:**
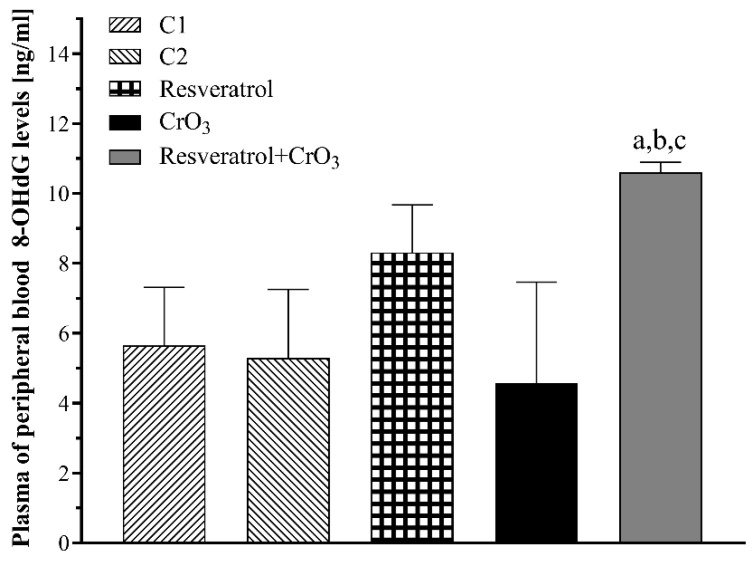
Effect of resveratrol and CrO_3_ on 8-hydroxydeoxyguanosine (8-OHdG, 7,8-dihydro-8-oxodeoxyguanosine) levels evaluated in peripheral blood plasma 48 h after treatments (*n* = 4 mice/group). Statistical significance was determined using one-way ANOVA followed by Tukey’s post-hoc test: ^a^ *p* < 0.012 vs. C1; ^b^ *p* < 0.007 vs. C2; ^c^ *p* < 0.002 vs. CrO_3_. C1, Control 1, vehicle only (distilled water); C2, Control 2, vehicle only (ethanol 30%); CrO_3_, chromium trioxide.

The 8-OHdG adduct was measured at 48 h in blood plasma since this method does not require sacrificing the animals. It is generally accepted that the excretion of the oxidized nucleosides 8-oxodG and 8-oxoGuo can be measured in fluids such as plasma, under the assumption that an organism maintains a steady-state with no changes in the rate of oxidation [25]. In that situation, the number of oxidized guanine moieties in the nucleic acid and its precursor pool must be equal to the number removed/excreted from the cell. Thus, the levels of 8-OHdG evaluated in fluids represent the balance between formation and repair rates. When evaluating oxidative damage to DNA using the 8-OHdG adduct repair levels in peripheral blood plasma analyzed by one-way ANOVA, there was a significant effect of treatment (*p* < 0.0015). The group treated with resveratrol prior to CrO_3_ (resveratrol + CrO_3_) had higher 8-OHdG levels than the control groups (C1, *p* < 0.012; C2, *p* < 0.007) and the CrO_3_ group (*p* < 0.002) (Figure 2). Although the resveratrol group displayed numerically higher 8-OHdG levels than the control C2 and CrO_3_ had lower levels than control C1, neither comparison was statistically significant.

### 2.3. Effect of Resveratrol and CrO_3_ on the Antioxidant System

**Figure 3:**
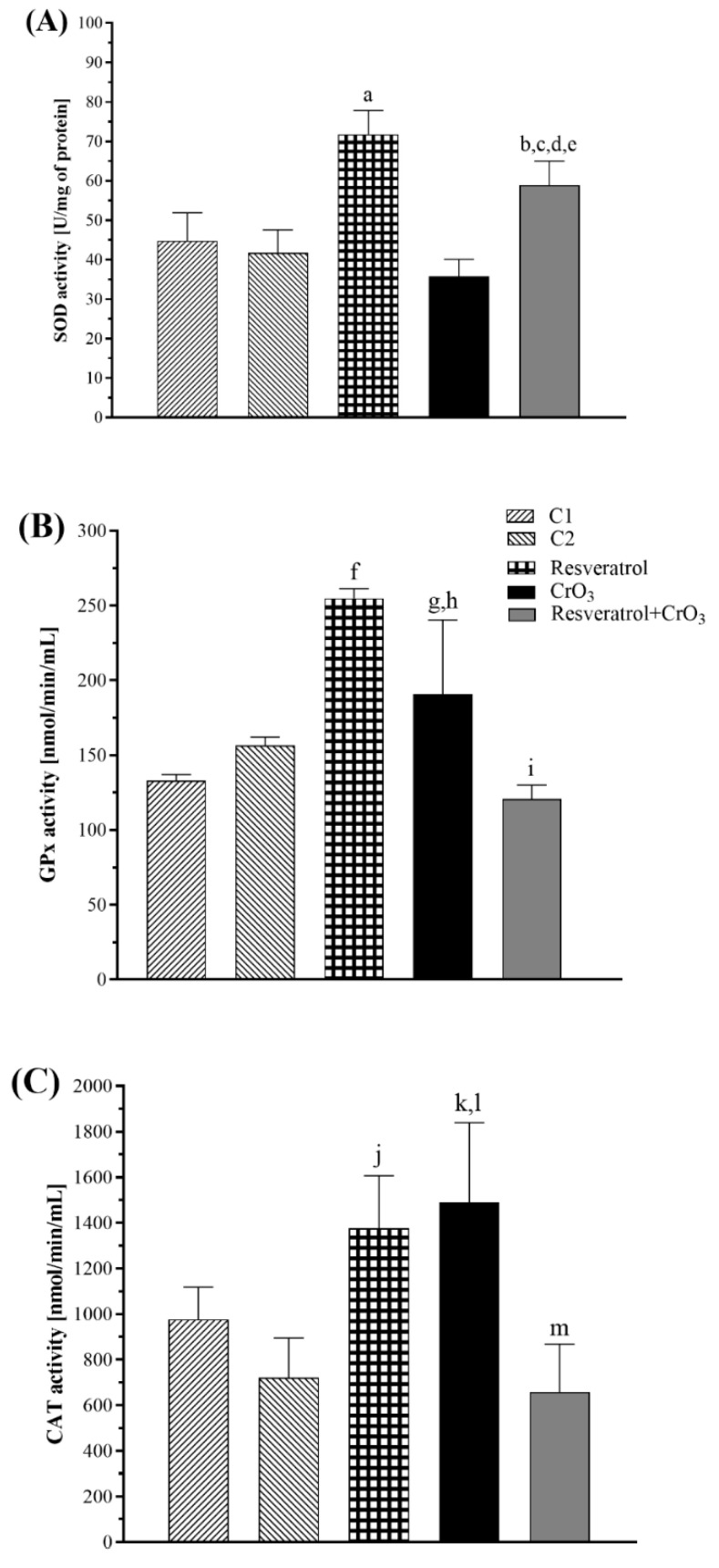
(**A**) Superoxide dismutase (SOD), (**B**) glutathione peroxidase (GPx), and (**C**) catalase (CAT) activities evaluated in peripheral blood at 48 h after treatments with resveratrol and CrO_3_ (*n* = 5 mice/group). Statistical significance was determined using one-way ANOVA followed by Tukey’s post-hoc test: ^a^ *p* < 0.0001 vs. C2; ^b^ *p* < 0.016 vs. C1; ^c^ *p* < 0.001 vs. C2; ^d^ *p* < 0.019 vs. resveratrol; ^e^ *p* < 0.0001 vs. CrO_3_; ^f^ *p* < 0.0001 vs. C2; ^g^ *p* < 0.019 vs. C1; ^h^ *p* < 0.004 vs. resveratrol + CrO_3_; ^i^ *p* < 0.0001 vs. resveratrol; ^j^ *p* < 0.005 vs. C2; ^k^ *p* < 0.034 vs. C1; ^l^ *p* < 0.0002 vs. resveratrol + CrO_3_; ^m^ *p* < 0.001 vs. resveratrol. C1, Control 1, vehicle only (distilled water); C2, Control 2, vehicle only (ethanol 30%); CrO_3_, chromium trioxide.

The effect of the treatments on the antioxidant system was determined by evaluating glutathione (GSH) levels and the enzymatic activity of superoxide dismutase (SOD), glutathione peroxidase (GPx), and catalase (CAT). During the reduction of Cr(VI) to trivalent chromium [Cr(III)] superoxide radical (O_2_^•^) is generated, which can be dismutated by SOD. While GPx and CAT, when interacting with hydrogen peroxide (H_2_O_2_), can inhibit the production of the hydroxyl radical (•OH), and GSH participates in one of the Cr(VI) reduction pathways [15]. The enzymatic activities of SOD, GPx, and CAT are shown in Figure 3. Data were analyzed with a one-way ANOVA. Treatment had a significant effect on SOD activity (*p* < 0.0001). The resveratrol group had higher SOD activity than the control group (C2) (*p* < 0.0001), and the resveratrol + CrO_3_ group showed an increase compared to the control groups (C1, *p* < 0.016; C2, *p* < 0.001), the resveratrol group (*p* < 0.019) and the CrO_3_ group (*p* < 0.0001). GPx activity was also significantly affected by treatment (*p* < 0.0001). The CrO_3_ treatment increased GPx activity compared to the control group C1 (*p* < 0.019) and the resveratrol + CrO_3_ group (*p* < 0.004). Resveratrol treatment alone also increased GPx activity compared to control group C2 (*p* < 0.0001). The resveratrol + CrO_3_ group had lower GPx activity than the resveratrol only group (*p* < 0.0001). There was also a significant effect of treatment on CAT activity (*p* < 0.0001). Resveratrol treatment increased CAT activity relative to the C2 control (*p* < 0.005), while treatment with CrO_3_ increased it compared to control group C1 (*p* < 0.034). CAT activity was lower in the resveratrol + CrO_3_ group than in the resveratrol group (*p* < 0.001) and the CrO_3_ group (*p* < 0.0002). Together, these results demonstrate that in the resveratrol + CrO_3_ mice, resveratrol restored GPx and CAT activity to levels similar to the controls. In the resveratrol + CrO_3_ group, SOD levels were higher than those of the control and CrO_3_ groups.

**Figure 4:**
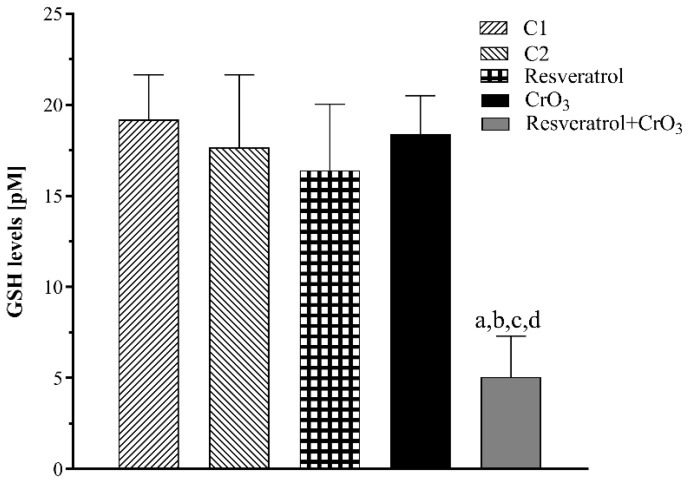
Average glutathione (GSH) levels evaluated in peripheral blood erythrocytes 48 h after treatment with resveratrol and CrO_3_ (*n* = 5 mice/group). Statistical significance was determined using one-way ANOVA followed by Tukey’s post-hoc test: ^a^ *p* < 0.0001 vs. C1; ^b^ *p* < 0.0001 vs. C2; ^c^ *p* < 0.0001 vs. resveratrol; ^d^ *p* < 0.0001 vs. CrO_3_. C1, Control 1, vehicle only (distilled water); C2, Control 2, vehicle only (ethanol 30%); CrO_3_, chromium trioxide.

The GSH levels are shown in Figure 4. There was a significant effect of treatment on GSH levels (*p* < 0.0001). There were no significant differences in GSH levels between CrO_3_-treated animals and any other group (*p* > 0.05), while resveratrol + CrO_3_ mice had significantly lower GSH levels than the controls (C1, *p* < 0.0001; C2, *p* < 0.0001), the resveratrol group (*p* < 0.0001), and the CrO_3_ only group (*p* < 0.0001).

### 2.4. Effect of Resveratrol and CrO_3_ on Apoptotic and Necrotic Cells

**Figure 5:**
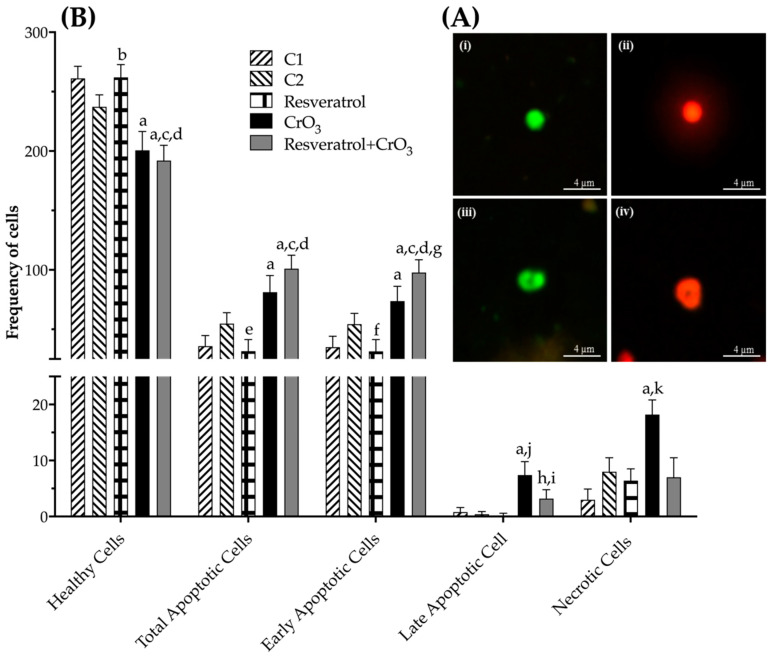
(**A**) Fluorescent microphotograph (400×) of peripheral blood cells using differential acridine orange/ethidium bromide (AO/EB) staining. (**i**) Healthy cell. (**ii**) Necrotic cell. (**iii**) Early apoptotic cell. (**iv**) Late apoptotic cell. (**B**) Effect of resveratrol and CrO_3_ on the frequencies of healthy, apoptotic (total, early, and late), and necrotic cells in peripheral blood, evaluated 48 h after treatments. A total of 300 nucleated cells were evaluated in each mouse (*n* = 5 mice/group). Statistical significance was determined using one-way ANOVA followed by Tukey’s post-hoc test: ^a^ *p* < 0.0001 vs. C1; ^b^ *p* < 0.03 vs. C2; ^c^ *p* < 0.0001 vs. C2; ^d^ *p* < 0.0001 vs. resveratrol; ^e^ *p* < 0.022 vs. C2; ^f^ *p* < 0.015 vs. C2; ^g^ *p* < 0.011 vs. CrO_3_; ^h^ *p* < 0.034 vs. C2; ^i^ *p* < 0.02 vs. resveratrol; ^j^ *p* < 0.001 vs. resveratrol + CrO_3_; ^k^ *p* < 0.0001 vs. resveratrol + CrO_3_. C1, Control 1, vehicle only (distilled water); C2, Control 2, vehicle only (ethanol 30%); CrO_3_, chromium trioxide.

Apoptotic and necrotic cells as well as cell viability were evaluated using differential acridine orange/ethidium bromide (AO/EB) staining (Figure 5A). The dual fluorochrome assay is capable of distinguishing between viable and nonviable cells based on membrane integrity. When cells are still viable, they keep the plasma membrane intact, allowing only AO to intercalate into DNA, which causes the nucleus to fluoresce green (Figure 5A(i,iii)). However, in nonviable cells, membrane integrity is lost, causing, ethidium bromide (EB) to also intercalate into DNA, making the nucleus fluoresce red since EB overwhelms AO staining (Figure 5A(ii,iv)). The color of the nucleus depends on the viability of the cell, not the state of the nucleus. Early apoptotic cells that have intact membranes but in which the DNA has begun to fragment still exhibit green nuclei because the EB cannot enter the cell, but chromatin condensation is visible as bright green patches in the nuclei (Figure 5A(iii)). As the cell progresses through the apoptotic pathway and membrane blebbing begins to occur, EB permeates the cell, producing a red-stained cell. Late apoptotic cells show bright red patches of condensed chromatin in the nuclei (Figure 5A(iv)); this distinguishes them from necrotic cells, which stain uniformly red (Figure 5A(ii)). When comparing the effect of treatments on apoptosis by one-way ANOVA, there was a significant effect of treatment on the frequency of healthy, total, early and late apoptotic cells as well as necrotic cells when compared to their control groups (*p* < 0.0001). Resveratrol reduced the frequency of total and early apoptotic cells compared to control groups (*p* < 0.022 and *p* < 0.015, respectively), while CrO_3_ induced an increased number of total, early, late apoptotic, and necrotic cells compared to control groups (*p* < 0.0001). In the resveratrol + CrO_3_ mice, there were fewer late apoptotic and necrotic cells compared to the CrO_3_ group (*p* < 0.001 and *p* < 0.0001, respectively) and an increase in total and early apoptotic cells compared to the control group (*p* < 0.0001) and the resveratrol group (*p* < 0.0001) (Figure 5B).

**Figure 6:**
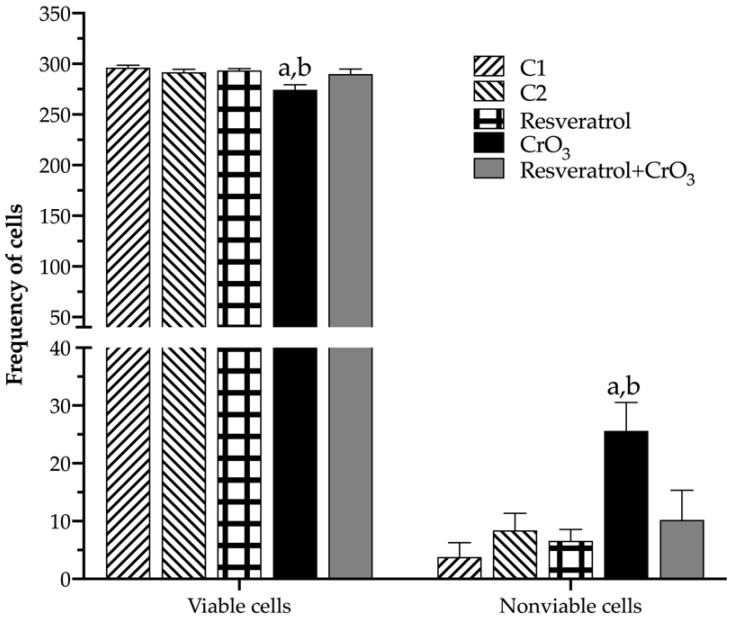
Effect of resveratrol and CrO_3_ on the frequencies of viable and nonviable cells in the peripheral blood of mice evaluated 48 h after treatment using differential acridine orange/ethidium bromide (AO/EB) staining. Viable cells include healthy and early apoptotic cells. Nonviable cells include late apoptotic and necrotic cells. A total of 300 nucleated cells were evaluated for each mouse (*n* = 5 mice/group). Statistical significance was determined using one-way ANOVA followed by Tukey’s multiple comparisons post hoc test: ^a^ *p* < 0.0001 vs. C1; ^b^ *p* < 0.0001 vs. resveratrol + CrO_3_. C1, Control 1, vehicle only (distilled water); C2, Control 2, vehicle only (ethanol 30%); CrO_3_, chromium trioxide.

Table 1 shows the PCE/NCE ratio. These evaluations were performed on the same samples and times used for MN. There were no significant effects in any of the treatments compared to their control groups (C1, C2) or time 0. However, when cell viability was compared in nucleated peripheral blood cells (48 h) using the dual fluorochrome assay, a significant effect of treatment on viable and nonviable cells (*p* < 0.0001) was observed (one-way ANOVA). The dual fluorochrome assay is an indicator of cell metabolism and death caused by cell membrane injury. Viable cells included those with an intact membrane, and thus they exhibited a nucleus fluoresced green by AO intercalation (healthy and early apoptotic cells; Figure 5A(i,iii), respectively). Moreover, nonviable cells included those in which the integrity of the membrane had been lost and that, therefore, presented a nucleus fluoresced red due to the intercalation of the EB (late apoptotic and necrotic cells; Figure 5A(ii,iv), respectively). Treatment with CrO_3_ increased nonviable cells compared to the control group (*p* < 0.0001), while treatment with resveratrol prior to CrO_3_ exposure decreased the nonviable cells observed in the group treated with CrO_3_ alone (*p* < 0.0001). Resveratrol treatment alone had no significant effect on cell viability (Figure 6).

The mice in the CrO_3_ group showed clinical signs of toxicity, including bristling hair, decreased mobility, and loss of appetite. The dose of 50 mg/kg of resveratrol did not exhibit any apparent clinical signs of toxicity. None of the mice exposed to resveratrol, CrO_3_, or both treatments died.

## 3. Discussion

The aim of this study was to (1) examine the protective effect of resveratrol against Cr(VI)-induced genotoxic damage in vivo and, (2) explore the possible protective pathways of resveratrol at the time of greatest induction of genotoxic damage by CrO_3_ (48 h). The evaluations were carried out on the same peripheral blood samples, in which the protection from genotoxic damage (MN) was studied. The pathways explored were adduct repair 8-OHdG levels, antioxidant system GSH levels, and enzymatic activities of SOD, GPx, and CAT. Our findings showed that the administration of resveratrol 4 h prior to exposure to CrO_3_ reduced the frequency of MN induced by this metal in Hsd:ICR male mice. Similarly, an approximation of the possible pathways involved in the protection of genotoxic damage induced by CrO_3_ was achieved.

To evaluate the genotoxic damage attributed to Cr(VI), a dose of 20 mg/kg CrO_3_ was administered intraperitoneally (ip). The 20 mg/kg dosage was based upon a previous study, in which this dose induced the formation of MN in the peripheral blood of mice [19,26]. Cr(VI) detoxification is relatively fast (no more than 48 h) when administered ip, in contrast to the effects observed with long-term oral and inhalation exposure to Cr(VI) [27]. Although the ip route is an artificial exposure route, it is useful for detecting genotoxic damage in short-term protocols, such as the MN assay, when testing compounds with potential clastogenic properties. Similarly, it is a more sensitive and direct route than inhalation or oral exposure [28,29]. Thus, a short-term protocol using the ip route of administration was selected to examine direct genotoxic damage induced by CrO_3_. Resveratrol bioavailability studies, which have described peak plasma concentrations from approximately 1 to 6 h after treatment [30], also support the use of a short-term protocol to assess genotoxic damage.

The increase in MN is an indication of the genotoxic effects exerted by Cr(VI). According to the guidelines of the Organization for Economic Cooperation and Development (OECD) and the Environmental Protection Agency (EPA), a substance or compound that induces more than 4 MN/1000 PCE should be considered a genotoxic agent [29,31]. Our results are consistent with the genotoxic damage reported for Cr(VI) compounds and particularly CrO_3_ [26,32], as increases greater than 5 MN were observed at all times evaluated. Administration of resveratrol 4 h before exposure to CrO_3_ reduced these frequencies of MN in vivo. When evaluating the levels of 8-OHdG in blood plasma at 48 h after CrO_3_ treatment, no significant alterations in adduct repair were detected. Notably, Maeng et al. [33] reported that inhalation of 18 mg/m^3^ of sodium chromate resulted in significantly elevated 8-OHdG levels in the lungs after 1 week. However, after 2 weeks of exposure, this dose produced no significant differences in pulmonary 8-OHdG levels, with full recovery after 3 weeks. Maeng et al. [33] also demonstrated that inhalation of higher sodium chromate levels did not significantly alter pulmonary 8-OHdG levels. Similarly, Thompson et al. [34] noted that the in vitro genotoxicity of Cr(VI) is primarily oxidative in nature at low concentrations. They observed that 8-OHdG reaches non-cytotoxic concentrations at 24 h in cell cultures treated with different doses of sodium dichromate. It is conceivable that ip administration of 20 mg/kg CrO_3_ might be too high to significantly affect 8-OHdG levels in the blood, in agreement with in vivo findings of Maeng et al. [33] and in vitro observations of Thompson et al. [34]. However, it should not be ruled out that Cr(VI) might reduce the levels of protein expression initiating DNA mismatch repair by inhibiting the hMLH1 and hMLH2 genes and the 8-oxoguanine DNA glycosylase1 (OGG1) repair enzyme involved in base excision repair (BER) [35,36,37]. Mice treated with resveratrol prior to CrO_3_ showed an elevation in 8-OHdG levels. There are the following two possible explanations for these results: (1) resveratrol activated repair mechanisms that counteract oxidative damage in DNA, and/or (2) resveratrol contributed to the elimination of 8-OHdG adducts formed by the oxidative damage. The 8-OHdG levels are known to be related to the balance between oxidative DNA damage and the rate at which it is repaired [25]. Yan et al. [38] observed in vitro that resveratrol activates the BER pathway, increasing the expression of OGG1. Further, Mikuła-Pietrasik et al. [39] noted that resveratrol enhanced the activity of the repair enzyme OGG1 in senescent human cells. In our study, resveratrol provided approximately 50% protection against genotoxic damage from CrO_3_ at all evaluation times. This effect might be attributed to the antioxidant properties of resveratrol, which enable this substance to interact with H_2_O_2_ and O_2_^•^ and •OH radicals [40]. Previously, Leonard et al. [41] demonstrated in vitro that resveratrol scavenged •OH in JB6 cells exposed to Cr(VI). In a previous study in vivo with (-)-epigallocatechin-3-gallate (EGCG), García-Rodríguez et al. [42] reported that 8-OHdG levels returned to control levels when EGCG and Cr(VI) were co-administered, contrasting with the findings of this study. They also found that co-administration of EGCG and Cr(VI) decreased the magnitude of MN increase compared to Cr(VI) alone [42], similar to the effect observed in this study with resveratrol. Hence, it is possible that when resveratrol was administered in combination with CrO_3_, the repair mechanisms were enhanced by this polyphenol, contributing to a reduction in MN levels.

In the group treated with resveratrol and CrO_3_, MN frequencies were reduced by 60, 51, and 46% at 24, 48, and 72 h, respectively. However, the reduction at 72 h was no longer significant. This may be due to the pharmacokinetics of CrO_3_. It has been reported that Cr(VI) compounds might be excreted within 48 h of exposure [27,43]. Hence, the greatest damage to DNA occurs during that period. Another possibility is that the micronucleated PCE induced over 24 h matured into NCE by 72 h, such that these were not quantified at that time. When leaving the bone marrow, PCE degrades ribosomal RNA in 24 h [44]. In other studies, this same trend was also observed in the reduction of MN at 72 h after administration of Cr(VI) [19,45].

The evaluations of SOD, GPx, CAT, and GSH activities were performed in peripheral blood samples obtained at 48 h because this did not require sacrificing the animals, which was necessary to continue with the evaluation of MN kinetics in the same individuals. Further, in vitro studies showed that resveratrol’s antioxidant properties may neutralize oxidative capacity in human erythrocytes [46], and SOD and GSH play important roles in the antioxidant system of erythrocytes [47]. In addition, altered functions of extracellular antioxidants may be assessed by the evaluation of antioxidant molecules in plasma [48]. SOD and GSH measurements were carried out in erythrocytes while CAT and GPx were carried out in plasma.

In the group treated with CrO_3_, there was a decrease in SOD activity accompanied by an elevation in GPx and CAT activities, which is consistent with previous findings. Both in vitro [49] and in vivo studies demonstrated the effects of oral [50] and ip [42] administration of Cr(VI) compounds on endogenous antioxidants, such as activities of SOD, CAT, GPx, heme oxygenase-1 (HO-1) and levels of GSH. Matés [51] proposed that SOD plays an important role as a first-line antioxidant defense enzyme by catalyzing the dismutation of O_2_^•^ to form H_2_O_2_, which is subsequently reduced to H_2_O by GPx and CAT. The decrease in SOD activity observed in the group treated with CrO_3_ might be related to its depletion by reacting with the O_2_^•^ radicals that are generated in excess during reduction to Cr(III). Meanwhile, the H_2_O_2_ generated by the activity of SOD may be increased due to the activity of GPx and CAT that was observed in this group. Regarding GSH levels, no significant changes were observed with CrO_3_ treatment, suggesting that reduction of Cr(VI) was not primarily mediated by this pathway. In this sense, in vivo studies noted that the reduction of Cr(VI) compounds is predominantly via nicotinamide adenine dinucleotide phosphate (NADPH) and other reducing agents such as ascorbate, cysteine, lipoic acid, fructose, and ribose [15]. In the group treated with resveratrol and CrO_3_, GSH levels were significantly decreased. GSH is a primary antioxidant molecule that plays a fundamental role in reducing cellular oxidative stress. GSH might act in the following different ways: (1) directly as an electron donor by eliminating O_2_^•^, (2) through GPx catalysis by reducing H_2_O_2_ levels, or (3) by forming complexes with detoxifying enzymes such as glutathione S-transferase (GST) [52]. GSH, GPx, and GST are some of the major antioxidant defense systems that scavenge ROS [53]. Resveratrol was found to induce phase II detoxification enzymes in in vitro and in vivo systems [54], and to increase GST activity in cultured aortic smooth muscle cells [55]. Further, several investigators have shown that polyphenols elevate GSH levels and stimulate the transcription of genes that are relevant for the synthesis of endogenous antioxidants, thus counteracting oxidative stress [56]. On the other hand, in this same group (resveratrol + CrO_3_), the activities of GPx and CAT were restored, and SOD activity increased. Data suggest that resveratrol might counteract CrO_3_-induced oxidative stress by an indirect antioxidant effect related to the regulation of the endogenous antioxidant system. In in vitro studies, Yao et al. [57] observed that resveratrol protected against oxidative damage induced by sodium sulfate dextran, while Chen et al. [8] found that polyphenols diminished damage mediated by sodium arsenite. In both cases, evidence indicated that the observed protection was related to increased SOD activity. SOD is a phase 2 enzyme that can be activated through the Nrf2/Keap1 signaling pathway. Nrf2 is a fundamental sensor of oxidative stress that plays a central role in the regulation of phase 2 antioxidant and detoxifying enzymes and related proteins [58]. Zhuang et al. [59] found that resveratrol regulates p-Nrf2 levels in a dose-dependent manner. This suggests that resveratrol attenuates the oxidative state, probably by activating the Nrf2 signaling pathway, which in turn elevates SOD activity. Resveratrol was also found to maintain the cellular redox balance by enhancing the activity of antioxidant enzymes, including HO-1, CAT, GPx, and SOD, in rat arterial endothelial cells [60]. Banu et al. [10] showed that 10 mg/kg resveratrol protected against potassium dichromate-induced oxidative stress in rat ovarian tissue by enhancing the activities of GPx, CAT, SOD, peroxiredoxin, and thioredoxin, and by lowering the concentration of H_2_O_2_. Therefore, it is possible that resveratrol also removed H_2_O_2_ generated by SOD activity, which reduced the need for GPx and CAT activation in the group treated with resveratrol and CrO_3_. Nevertheless, it is important to keep in mind that the endogenous antioxidant system is dynamic, and thus, it is possible that our results may depend upon the evaluation time (48 h), the dose of resveratrol used, and the experimental model.

Although the PCE/NCE ratio is included in the MN assay as an indicator of cytotoxicity [29], no marked changes in the PCE/NCE ratio were observed in any of the treated groups in this study. These results need to be interpreted with caution, since when toxicity occurs during erythropoiesis, activation of cell division mechanisms may mask the effects on the PCE/NCE ratio [61]. For this reason, cytotoxicity was assessed by analyzing cell viability using the AO/EB differential staining technique, which allows us to distinguish between viable and non-viable cells according to the integrity of the membrane [45]. When cell viability, apoptosis, and necrosis were determined, treatment with CrO_3_ significantly increased the numbers in total, early and late apoptotic cells, as well as necrotic and nonviable cells. These results corroborate the cytotoxicity reported for Cr(VI) compounds [26,27,45]. ROS generation and DNA damage induced by Cr(VI) exposure might play an essential role in cytotoxicity and the apoptotic signaling pathway [62]. It has been proposed that apoptosis induction is mediated by DNA damage sensors that directly activate p53 through proteins such as DNA-dependent kinase (DNA-PK) or indirectly through mutated ataxia-telangiectasia (ATM) and ATM-Rad3 (ATR) with Chk1 or Chk2 [63]. It has also been reported that apoptosis induction may be mediated through p53-independent pathways such as cleaved caspase 3 and cytochrome C [10,64]. However, when polyphenol was administered 4 h prior to CrO_3_ in mice, a significant increase in early apoptotic cell number was observed compared to the group treated with CrO_3_ alone. This effect was masked when early and late apoptotic cells were summed, as late apoptotic cells also showed a significant decrease in this group. Hence, it is possible that the enhanced induction of early apoptotic cells following combined CrO_3_ resveratrol treatment may contribute to the elimination of cells containing Cr(VI)-induced DNA (MN) damage. Although there are no apparent studies of the effects of resveratrol on Cr(VI)-induced apoptosis pathways, resveratrol was reported to induce apoptosis as a mechanism of elimination of damaged cells in cancer cell lines [4]. Further, alterations in the expression of the Bcl2 protein, the loss of mitochondrial function, the release of cytochrome c, and the activation of caspases trigger the response for the activation of apoptosis [65]. Mirzapur et al. [66] reported that in breast cancer cells, resveratrol elevated the levels of the Bcl2/Bax protein, as well as the expression of p53 genes and caspases 3 and 8. Therefore, based upon these observations and the results reported in the present study, there is clearly a need to conduct studies that aim to reach a more detailed understanding of how resveratrol interacts with proteins such as p53, DNA-PK, ATM, ATR, Bax, Bcl2, caspases (3 and 8), among others. Indeed, these studies may greatly contribute toward understanding the mechanisms by which polyphenols such as resveratrol might contribute to the elimination of cells with genotoxic damage induced by compounds with carcinogenic potential such as Cr(VI).

The administration of resveratrol reduced the frequency of MN induced by CrO_3_ and resveratrol treatment itself did not produce DNA damage (MN induction). The reduction in GSH and elevation in apoptotic cell number with both treatments (resveratrol + CrO_3_), as well as increases in SOD, GPx, CAT, and 8-OHdG (the latter non-significant) with resveratrol alone, suggest a toxic effect. In in vitro and in vivo studies, it has been observed that resveratrol exhibits biphasic effects (antioxidant and prooxidant). Meira-Martin et al. [67] considered that the increases in SOD and SOD/CAT activity observed in vitro with different doses of resveratrol are generated to maintain the cellular redox balance. Hence, it has been proposed that its prooxidant activity contributes to the activation of the endogenous antioxidant system [68]. Sinha et al. [69] found that the prooxidative effects of resveratrol are associated with the generation of the O_2_^•^ radical, H_2_O_2_, and a complex mixture of semiquinones and quinones. However, in this study, no marked effects on viable cell numbers were observed in the group treated with resveratrol and CrO_3_, and this group exhibited a significant decrease in necrotic cell frequency, suggesting that polyphenols diminished the cytotoxicity produced by CrO_3_. Other in vivo studies also noted that resveratrol diminished the toxicity induced by metals, such as arsenic [70], copper, and zinc [71], contributing to the balance of the cellular redox system and reducing the expression of proinflammatory cytokines. On the other hand, the administration of resveratrol alone significantly decreased total and early apoptotic cells when compared to its control, suggesting that resveratrol alone does not induce toxicity and that it reduced the potential toxic effect of the vehicle (30% ethanol). Although ethanol is a less toxic polar vehicle than other vehicles such as dimethyl sulfoxide (DMSO) [72], ad libitum administration (11%) was shown to increase serum ROS in treated mice for 60 days [73]. Based on our results, it is suggested to extend these studies by using more diluted doses of resveratrol and even administering it in repeated doses, to reduce the possible toxic effects and improve the protection against the genotoxic damage observed in the present study.

## 4. Materials and Methods

### 4.1. Chemicals and Reagents

Cr(VI) [CrO_3_, purity grade 99.9%; CAS 1333-82-0], 3,4′,5-trihydroxy-trans-stilbene [resveratrol, purity grade ≥ 98%; CAS 501-36-0], AO [CAS 10127-02-3], and EB [CAS 1239-45-8] were obtained from Sigma Chemical Co. (St. Louis, MO, USA).

### 4.2. Animals

A group of 25 adult male Hsd:ICR mice (8–12 weeks old, 28–35 g) were used in the experiment. The animals were obtained from Harlan^®^ (Mexico City, CDMX, Mexico) at the “Facultad de Química, Universidad Nacional Autónoma de México-UNAM” and acclimated for two weeks prior to initiating the experiments. During the acclimation period, the groups of five mice were kept in a plastic cage at a controlled room temperature (22 ± 2 °C) with a 12-h light-dark cycle (the lights came on at 7:00 a.m. and went off at 7:00 p.m.). Mice had free access to food (Purina-Mexico^®^, Mexico City, CDMX, Mexico; small rodent chow) and water. Considering that in previous studies there were no differences between males and females in the genotoxic effects of CrO_3_ administered by ip injection [16,26,45], this study was carried out using only male mice, in accordance with guidelines for the testing of chemicals (mammalian erythrocyte micronucleus test) of the OECD and the EPA [29,31].

The mice were randomly divided into five groups of five individuals each. Two control groups were used (C1: mice treated ip with sterile distilled water and C2: mice treated with 30% ethanol by gavage) because the CrO_3_ solution was prepared by dissolving the compound in water, whereas resveratrol was dissolved in 30% ethanol. The resveratrol group was treated with a single dose of 50 mg/kg by gavage, and the CrO_3_ group was treated with a single dose of 20 mg/kg ip. The last group received combined resveratrol and Cr(VI) treatments (resveratrol + CrO_3_); the mice were treated with resveratrol at 50 mg/kg by gavage 4 h prior to CrO_3_ ip injection (20 mg/kg).

The assessment was carried out on peripheral blood obtained from the tail vein since this does not require animals to be sacrificed.

### 4.3. Micronuclei Assay

For the MN evaluations, sequential peripheral blood samples (5 μL) were obtained from the same individuals (0 to 72 h), and 0–h samples were designated as a negative control. The samples were placed directly onto slides previously treated with AO, as described by Hayashi et al. [74]. Two slides were prepared for each mouse and were stored in the dark at 4 °C for 24 h. The assessments were performed by identifying PCE, NCE, and MN in PCE using a fluorescence microscope (Nikon^TM^ OPTIPHOT-2; Tokyo, Japan) with blue excitation (480 nm) and a barrier filter emission (515–530 nm) at 100× magnification. MN analysis was based upon 4000 PCE per mouse, and the presence of MN was considered to indicate genotoxic damage. The relative proportion of PCE to NCE was also analyzed for 2000 erythrocytes.

In this study, underlying metabolic processes such as 8-OHdG adduct repair, endogenous antioxidant component system, apoptosis, and cell viability analyses were also evaluated because these processes may be involved in preventing Cr(VI)-induced DNA damage. These parameters were measured using the same peripheral blood samples obtained at 48 h after treatments, in which MN were measured since this is the time when the greatest genotoxic damage induced by Cr(VI) has been observed [26].

### 4.4. Plasma 8-Hydroxydeoxyguanosine Levels

Plasma 8-OHdG levels were determined using an enzyme-linked immunosorbent assay. Peripheral blood samples (50 µL) were centrifuged (15 min at 2500× *g*) at room temperature. The plasma was collected and immediately analyzed according to the manufacturer’s instructions using Trevigen’s HT 8-oxo-dG ELISA Kit II (No. 4380-192-K; Gaithersburg, MD, USA). The absorbance at 450 nm of each well was determined using a Multiskan™ FC microplate reader (Thermo Scientific™, Vantaa, Finland). Product formation is inversely proportional to the amount of 8-OHdG present in the sample. The 8-OHdG levels were determined in duplicate (per sample) and according to the 8-OHdG standard curve.

### 4.5. Antioxidant System

The antioxidant system was evaluated by determining the activities of SOD, GPx, and CAT, as well as the GSH levels.

#### 4.5.1. Superoxide Dismutase Activity

SOD activity was evaluated in peripheral blood erythrocytes. Fifty-µL samples were diluted in phosphate-buffered saline. The erythrocytes were separated using FicollPaque™ (Sigma Chemical Co., St. Louis, MO, USA). (800× *g* for 25 min at 12 °C). The precipitate was separated, and cold distilled water (4 °C) was added (10:1). Then, it was incubated (0 °C for 15 min) to lyse. Hemoglobin was then precipitated by adding ethanol and chloroform (10,000× *g* for 10 min at 4 °C). The SOD activity was determined according to the manufacturer’s instructions using Trevigen’s HT Superoxide Dismutase Assay Kit (No. 7501-500-K; Gaithersburg, MD, USA). The absorbance was read at 450 nm at 1-min intervals for 10 min in a Multiskan™ FC microplate reader (Thermo Scientific™, Vantaa, Finland). One unit of SOD activity was defined as the amount of protein that inhibited tetrazolium salt (WST-1)-formazan, up to a maximum of 50%. The protein concentration was determined according to the instructions for the Cayman Chemicals Protein Determination Kit (No. 704002; Ann Arbor, MI, USA). SOD activity was measured in duplicate (per sample) and according to the SOD standard curve.

#### 4.5.2. Glutathione Peroxidase Activity

GPx activity was detected in the plasma of peripheral blood. Twenty-five µL samples were centrifuged at 1000× *g* for 10 min at 4 °C. The plasma was collected and diluted with the GPx sample buffer (1:2) included in the kit. GPx activity was determined according to the instructions for the Cayman Chemicals Glutathione Peroxidase Assay Kit (No. 703102; Ann Arbor, MI, USA). One unit of GPx was defined as the amount of enzyme that oxidized 1 nmol of NADPH/min. The absorbance was read at 340 nm in a Multiskan™ FC microplate reader (Thermo Scientific™, Vantaa, Finland). GPx activity was determined in duplicate (per sample) and according to the GPx standard curve.

#### 4.5.3. Catalase Activity

CAT activity was evaluated in the plasma of peripheral blood. Twenty-five µL samples were centrifuged at 1000× *g* for 10 min at 4 °C to obtain plasma. The activity of CAT was evaluated according to the instructions for the Cayman Chemicals Catalase Assay (No. 707002; Ann Arbor, MI, USA). This kit uses the peroxidation function of CAT to determine enzyme activity. The absorbance was read at 540 nm in a Multiskan™ FC microplate reader (Thermo Scientific™, Vantaa, Finland). One unit of CAT was defined as the amount of enzyme that induced the formation of 1 nmol of formaldehyde/min. CAT activity was measured in duplicate (per sample) and according to the CAT standard curve.

#### 4.5.4. Glutathione Levels

GSH levels were evaluated in the erythrocytes of peripheral blood. Fifty µL samples were centrifuged at 3000× *g* for 15 min at 0 °C. The erythrocytes were suspended in 5% cold (*w*/*v*) metaphosphoric acid, mixed and stored at 0 °C for 15 min. Subsequently, the suspension was centrifuged at 14,000× *g* for 10 min at 4 °C. The clarified supernatant was collected, and GSH levels were analyzed according to the instructions using Trevigen’s HT Glutathione Assay Kit (Item No. 7511-100-K; Gaithersburg, MD, USA). The absorbance was read at 405 nm in a Multiskan™ FC microplate reader (Thermo Scientific™, Vantaa, Finland). The levels of GSH were determined in duplicate (per sample) according to the GSH standard curve.

### 4.6. Apoptosis and Cell Viability

To evaluate apoptosis, necrosis, and cell viability, differential acridine AO/EB staining was performed using a technique previously adapted for peripheral blood [45]. Ten µL samples were centrifuged at 4500× *g* for 5 min. The cell pellet was resuspended in 20 μL of AO/EB dye mix and plated on a clean slide. Two slides were prepared per mouse, and the analysis was performed immediately. The assessments were based upon 300 cells per mouse. Apoptotic, necrotic, viable, and nonviable cells were identified using a fluorescence microscope OPTIPHOT-2 (Nikon^TM^; Tokyo, Japan) with blue excitation (480 nm) and a barrier filter emission (515–530 nm) at 40× magnification.

### 4.7. Statistical Analysis

Each mouse was considered an independent replicate according to the OECD and EPA guidelines [29,31]. Individual samples were averaged for each experimental group. The MN frequencies, PCE/NCE ratio, viability (viable/nonviable cells), number of apoptotic and necrotic cells, levels of 8-OHdG and GSH, and activities of SOD, GPx, and CAT are expressed as the mean ± standard deviation (SD). The data were checked for normality using the Shapiro–Wilk test. Statistical significance between the groups for MN was determined by using two-way RM-ANOVA because MN depends on two factors (i.e., treatment and time). The treatment is independent, while the evaluations at each time are considered dependent since the samples were obtained from the same mouse. For the other parameters, one-way ANOVA was used because the evaluations depended only on one factor (i.e., treatment). In the analysis of ANOVA, post hoc Tukey multiple comparisons were carried out. GraphPad Prism 8.0 (GraphPad Software, San Diego, CA, USA) was used for all analyses. Differences were considered significant at *p* < 0.05.

## 5. Conclusions

Our findings demonstrate a protective effect of resveratrol against Cr(VI)-induced genotoxic damage, by reducing the frequency of MN induced by CrO_3_ in vivo. Likewise, an approximation of the possible pathways involved in the protection of genotoxic damage induced by these compounds with carcinogenic potential, such as Cr(VI), was achieved. Resveratrol showed effects on the modulation of the endogenous antioxidant system, 8-OHdG adduct repair, and apoptosis when administered 4 h prior to Cr(VI) exposure. These effects suggest that these pathways might be involved in the protection provided by this polyphenol against genotoxic damage induced by Cr(VI). Although resveratrol treatment modified endogenous antioxidant system constituents, the dose of 50 mg/kg alone did not alter MN frequencies, suggesting that it is not related to the induction of DNA damage. In vivo studies using more diluted doses of resveratrol and even administering it in repeated doses, as well as direct evaluations in target organs, could help determine the specific mechanisms, by which resveratrol counteracts Cr(VI)-induced genotoxicity. These studies contribute to the understanding of the potential antigenotoxic value of polyphenols such as resveratrol, and to the exploration of their possible use as chemotherapeutic agents in the prevention and treatment of diseases related to genotoxic damage.

## Figures and Tables

**Table 1:** PCE/NCE ratio in peripheral blood of mice treated with resveratrol and CrO_3_.

Treatment	Dose (mg/kg)	Time Analysis (h)	n	PCE/NCE 1000 Cells (mean ± SD)
C1	0	0	5	48.5 ± 8.6
		24		46.7 ± 4.6
		48		48.2 ±11.9
		72		52.0 ± 8.2
C2	60	0	5	46.7 ± 7.0
		24		47.2 ± 9.6
		48		53.0 ± 7.5
		72		43.4 ±13.2
Resveratrol	50	0	5	54.8 ±12.2
		24		47.5 ± 9.5
		48		48.7 ± 5.9
		72		50.5 ± 6.4
CrO_3_	20	0	5	46.5 ±10.0
		24		45.8 ± 7.0
		48		51.1 ±12.8
		72		39.8 ±12.9
Resveratrol + CrO_3_	50 + 20	0	5	45.6 ± 7.9
		24		46.4 ± 2.8
		48		53.8 ±12.7
		72		44.6 ± 5.8

A total of 2000 erythrocytes were evaluated in each mouse (*n* = 5 mice/group). C1, Control 1, vehicle only (distilled water); C2, Control 2, vehicle only (ethanol 30%). CrO_3_, chromium trioxide; PCE, polychromatic erythrocytes; NCE, normochromatic erythrocytes.

## Data Availability

The data presented in this study are available on request from the corresponding author.

## Sample Availability

Samples of the compounds are not available from the authors.
